# Comparative analysis of physiological traits and gene expression patterns in nitrogen deficiency among barley cultivars

**DOI:** 10.1186/s43141-023-00567-w

**Published:** 2023-11-10

**Authors:** Zohreh Hajibarat, Abbas Saidi, Habibollah Ghazvini, Zahra Hajibarat

**Affiliations:** 1 Department of Plant Sciences and Biotechnology, Faculty of Life Sciences and Biotechnology, Shahid Beheshti University, Tehran, Iran; 2 Department of Cearal Research, Seed and Plant Improvement Institute (SPII), Agricultural Research, Education and Extension Organization (AREEO), Karaj, Iran

**Keywords:** Barley, Nitrogen deficiency, Nitrate transporter genes, Physiological traits

## Abstract

**Background:**

Nitrogen is one of the most important mineral nutrients for plants and is absorbed by the root system mainly in the inorganic form (NH^+^_4_ and NO^−^_3_). Plants absorb nitrogen as a food source for growth, biomass production, and development. Nitrogen is mainly absorbed as nitrate, which is the most common source of nitrogen available to higher plants. One of the unique features of nitrate transport is that NO^−^_3_ is both a substrate for transport and an inducer of NO^−^_3_ transport systems in genes and at physiological levels.

**Methods:**

In the present study, morphological and physiological traits (chlorophyll a/b, total chlorophyll, and carotenoid, antioxidant enzymes, and protein content), correlation between traits and gene expression, and principle component analysis of traits among five barley cultivars were measured in response to nitrogen deficiency (ND). The starved plants were transferred to a nutrient solution containing 0.2 mM and 2 mM NO^−^_3_ up to 7 and 14 days after ND application and non-stressed conditions, respectively.

**Results:**

Gene expression analysis revealed that the 10 *HvNRT2* genes were induced in the leaf and root tissues at 7 and 14 days after ND treatments in five barley cultivars. Expression of *NRT2* genes by relative quantitative qRT-PCR analysis for 10 *HvNRT2* genes were determined. Based on the gene expression, *HvNRT2.1*, *HvNRT2.2*, and *HvNRT2.4* were strongly induced by NO^−^3, peaking at 7 and 14 days after ND treatment. In contrast, the *HvNRT2.4* showed only moderate induction in both leaves and roots. From our results, the Reyhan cultivar showed a significant increase in root fresh weight (RFW), protein content, and antioxidant enzyme activity in roots at 7 and 14 days after ND treatment as compared to the non-stressed condition. A highly positive correlation was observed between root catalase (CATr) and HvNRT2.2/2.5/2.6 leaves.

**Conclusion:**

The expression of HvNRT2.4 is increased during long-term nitrogen starvation, while the expression of *HvNRT2.1* and *HvNRT2.2* are transiently increased by ND. Based on physiological and morphological traits and molecular mechanisms, the Reyhan is considered a tolerant cultivar under ND condition.

## Background

Nitrogen plays an essential role in the regulation of several biological processes such as carbon metabolism, amino acid metabolism, and protein synthesis. Nitrogen starvation affects several major physiological and biological processes that affect growth and development [1], root architecture [2], lignin content [3], anthocyanin, phosphorus, potassium content [4], chlorophyll synthesis [5], and CO_2_ uptake in plants [6]. Nitrogen (N) is an essential plant growth nutrient whose coordinated distribution from source to sink organs is crucial for seed development and overall crop yield. Barley, Hordeum vulgar, is one of the most important staple food grains from the Poaceae family. However, grain yield is limited by a number of stresses. Among these stresses, insufficient use of nitrogen is the most important.

Selection of tolerant cultivars to abiotic stress has become one of the main goals of adaptation. In previous reports, the response, adaptation, genotype differences, and molecular mechanisms under LN stress have been comprehensively studied in such plants as *Arabidopsis* [7], rice [8], maize [9], and wheat [10]. Among the different forms of nitrogen, nitrate (NO^−^3) is the main absorbed form in most agricultural soils and is preferentially absorbed by most cereals except paddy rice, where the ammonium form (NH^+^_4_) is preferred [11]. One of the most important consequences of abiotic stress is the imbalance between the production of reactive oxygen species (ROS) and antioxidant defense systems (AOX), which can lead to cell loss and cell death. Catalase (CAT), peroxidase (POD), and ascorbate peroxidase (APX) are considered as the main antioxidant enzymes to inhibit ROS, and the inhibition ability is related to the activity of antioxidant enzymes in plants [12]. Nitrate is often limited in availability in most agricultural soils with significant temporal and spatial fluctuations. As sessile organisms, plants must be able to rapidly adapt to these changing soil nitrate concentrations to optimize nitrogen uptake. In order to increase the ability of plants to absorb N fertilizer, it is important to understand the processes by which plants acquire nitrate and how to regulate this process [13, 14]. Most plants have two types of nitrate uptake systems with associated transporter genes. Low-affinity transport system (LATS) when nitrate availability is > 0.5 mM and high-affinity transport system (HATS) when external nitrate availability is < 0.5 mM [15]. Depending on the external NO^−^_3_ concentration, low-affinity transport systems (LATS) and high-affinity transport systems (HATS) affect NO^−^_3_ uptake in plants under non-stressed conditions [16, 17]. When nitrate enters the plant, it is assimilated into amino acids in a series of reactions facilitated by a known set of enzymes, although when sufficient nitrate is available, it can be temporarily stored in vacuoles before absorption. Nitrogen compounds formed during uptake are stored in source tissues (e.g., leaves in cereals) during vegetative growth and then transferred to sink during the reproductive stage (e.g., seed development in cereals) in a process called nitrogen remobilization. The NRT2 gene family, belonging to HATS, was shown to play an important role in NO^−^_3_ uptake from soil under nitrogen-limited conditions [18]. However, the temporal transcriptional expression patterns of NRT1 and NRT2 gene families and their effects on nitrogen uptake in wheat have been unclear for a long time. Nevertheless, it is clear that the NO_3_ transport capabilities of most NRT2 proteins in plants require the involvement of a chaperone protein, NAR2, as part of a two-component high-affinity NO_3_ uptake system [19, 20]. Nitrogen use efficiency (NUE) is a complex trait that involves several molecular, biochemical, and physiological processes [21]. Improved NUE often results in increased aboveground biomass, seed yield, protein content, and overall economic performance [22].

The AtNRT2.1 is a component of the high-affinity nitrate uptake system that functions as a nitrate sensor or signal transducer downstream of the actual sensor, independent of its role in nitrate uptake [23]. The OsNRT2.1 and OsNRT2.2 are expressed in all root cell types with stronger activity in the epidermis in rice, and OsNRT2.3 expression is detectable in the root and lateral root [24, 25]. The expression of OsNRT2.4 is limited in the lateral root region and to a lesser extent in the companion cells of the crown root. A pervious study showed that osnrt2.4 mutants show defects in lateral root length and number under both high and low nitrate regimes [26]. It was found that different cultivars tolerant to low N showed significant differences in N metabolism enzyme activity, dry matter weight, etc., which should be considered in further research [27]. Therefore, in the present study, we investigated the effects of ND on chlorophyll content, protein content, dry and wet weights of roots and leaves, antioxidant enzymes, and the expression of transporter genes of barley cultivars with different ND tolerance. We compared high and low N use efficiency (NUE) barley cultivars.

## Methods

Ten (10) seeds of five barley cultivars were germinated on wet Whatman filter papers in Petri dishes. Seven- to 10-day-old seedlings, seedlings with a uniform growth state, were moved to 10-L containers. The plants were treated with a modified Hoagland nutrient solution [28] 2 mmol/L NH_4_NO^−^_3_, 0.4 mmol/L MgSO_4_, 0.3, mmol/L K_2_SO_4_, 0.2 mmol/L KH_2_PO_4_, 0.4 mmol/L CaCl_2_, 0.19 μmol/L CuSO^4^, 46.9 μmol/L H3BO3, 4.5 μmol/L MnCl_2_, 1 μmol/ L Na_2_MoO_4_, 0.38 μmol/L ZnSO_4_, and 19.9 μmol/L Fe (III) EDTA. The pH of the solution was adjusted to 5.8 with NaOH. The two N treatments included 0.2 mmol/L NH_4_NO^−^_3_ (ND) and 2 mmol/L NH_4_NO^−^_3_ (as non-stressed) conditions [29]. After 7 and 14 days of ND treatments, plant height (PH), leaf dry weight (LDW), root dry weight (RDW), leaf fresh weight (LFW), root fresh weight (RFW), and root lengths (RL) were recorded. Plants with uniform growth status were subsequently harvested as replicates, separated into roots and shoots, and dried in the oven at 72 °C for 3 days to obtain RDW and LDW.

### Characterization of physiological indices

To investigate physiological traits, ND and non-stressed treatments were applied to 7- to 10-day-old seedlings. Samples from non-stressed and stressed barley leaves and root were collected at 7 and 14 days after ND application. The fresh leaf samples were washed by distilled water in the laboratory. Then, they were left to dry at room temperature (18 °C) to be analyzed for the determination of chlorophylls (Cha and Chb) and carotenoid contents. Accurately weighted 0.5 g of fresh plant leaf and root samples were taken and homogenized in tissue homogenizer with 10 ml of acetone extracting solvent. The homogenized samples were centrifuged at 12,000 rpm for 15 min at 4 °C. The supernatant was separated and 1 ml of it was mixed with 4 ml of the acetone solvent. The solution mixture was analyzed for Chlorophyll-a, Chlorophyll-b, and carotenoid contents in a spectrophotometer. The activity of antioxidant enzymes, including polyphenol oxidase (PPO), catalase (CAT), and peroxidase (POD), was measured using the spectrophotometric method (BIORAD, SmartspecTM plus-USA) according to Kar and Mishra method [30].

### Protein extraction

To analyze protein content, ND and non-stressed treatments were applied to 7- to 10-day-old seedlings. Samples from non-stressed and stressed barley leaves were collected at 7 and 14 days after ND application. Preparation of protein and enzyme extract were carried out following the method [31]. In brief, fresh leaves (0.5 g) were ground to a fine powder in liquid nitrogen using a pre-chilled mortar and pestle and then extracted in 3 mL of 0.2 M potassium phosphate buffer, pH 7.0, containing 0.1 mM ethylenediaminetetraacetic acid (EDTA). The extract was centrifuged for 20 min at 13,000 rpm and 4 °C, and the supernatant was used for protein activity assay. The total protein content for all samples was determined by the method of Bradford [32] using bovine serum albumin as a standard.

### RNA extraction and expression pattern of nitrate transporter genes

For the preparation of RNA obtained from leaf and root tissues, samples were collected separately from barley seedlings under ND stress and non-stressed conditions. Sampling was done at 7 and 14 days after ND treatment. Total RNA was extracted from nitrogen-deficient and non-stressed leaves and roots using the RNX-Plus kit (Sinaclone) according to the manufacturer’s instructions. The purity and concentration of RNA were determined by NanoDrop and its quality was confirmed using 1% agarose gel analysis. Then, the cDNA synthesis was performed according to the instructions of the cDNA synthesis kit. Three repetitions were performed to analyze each gene, where the actin gene was used as a reference gene. All primers used in the gene expression analysis were designed using the Oligo program (Table 1). Gene expression was performed with a real-time instrument using Cybergreen as described in the manufacturer’s instructions. For the relative expression of genes through 2^−∆∆CT^ after normalization, the value of Ct for nitrate transporter genes was determined using actin as a reference gene. The qRT-PCR analysis was performed to determine the expression profile of *HvNRT2.1*, *HvNRT2.2*, *HvNRT2.3*, *HvNRT2.4*, *HvNRT2.5*, *HvNRT2.6*, *HvNRT2.8*, *HvNRT2.9*, and *HvNRT2.11* genes using leaf and root tissues under non-stressed and ND treatments. Results are discussed in relation to distinct roles for individual NRT2 family members. The SPSS software was used for expression analysis, physiological, and morphological traits. The TBtools was utilized to draw the heatmap which was used to display the differential expression of genes and the correlation between the physiological traits and gene expression. Statistical analyses were performed using SPSS version 20.0 statistical software. The significant variations between means were compared at *P* < 0.05 (Duncan’s test). Statistical graphs were generated using GraphPad Prism version 9 software and the statistical significance was defined at *P* < 0.05.

**Table 1:** The primer sequences of *HvNRT2* gene used in this study

Primer name	Primer sequence
HvNRT2.1F	GCTCCGCATGTTGATGTTTA
HvNRT2.1R	TGACGTTGCCGTTTGACTTA
HvNRT2.2F	TCATCTGTCTGCAGGAATCG
HvNRT2.2R	CCACATGTAACTGCGCGTAT
HvNRT2.3F	CATATCGCAGGCCAAAAGTT
HvNRT2.3R	CCTTATACGTGCTGGGGTGT
HvNRT2.4F	CCAGCACGTATGAGACTGGA
HvNRT2.4R	ACGCCTTATTACAGCCGATG
HvNRT2.5F	TGGACCGAGGAGGAGCGT
HvNRT2.5R	GGAGCTCTTCGGACCTCACAC
HvNRT2.6F	CATGCACGCTGCCCGTCGCT
HvNRT2.6R	GTCTCATACGTGCTGGGGCG
HvNRT2.7F	AGTACTACGGTGCCGAGTGG
HvNRT2.7R	GTTTGGTGGGCTGGTAGGTA
HvNRT2.8F	GACCGAGGAGGACTACTACGC
HvNRT2.8R	CGTGTACGGTAGGGAAGTAG
HvNRT2.9F	TCCATGCTCCTCCCACCCA
HvNRT2.9R	CGTGTACGGTAGGGAAGTAG
HvNRT2.11F	CTGCATCAGGGCTTACCTTC
HvNRT2.11R	CGGAGGGAGTAGGTTGGTAAA
HvActin F	GGTCCATCCTAGCCTCACTC
HvActin R	GATAACAGCAGTGGAGCGCT

## Results

Among the morphological traits measured at 7 and 14 days after applying ND, only the Mahtab cultivar showed a significant decrease in LDW at 7 days after ND application. On the other hand, the Mahtab cultivars showed a significant decrease in RDW at 7 days after ND application, but Bahrokh revealed a significant increase in RDW at 7 days after ND application. The RFW was significantly decreased in the Mahtab cultivar at 7 days after ND application as compared to the non-stressed condition whereas, the RFW was significantly increased in the Reyhan cultivar at 14 days after ND application as compared to the non-stressed condition. No significant differences were observed in the leaf fresh weight, plant height, and root length at 7 and 14 days after ND application (Table 2).

**Table 2:** Comparison of means for morphological traits measured (RDW, LDW, and RFW) in five barley cultivars

Cultivars	Treatments	RDW	LDW	RFW
Behrokh	S7	28a	23.6 abcdef	174.5 abcd
Behrokh	N7	14.7bcdefg	20.3 bcdef	133.2 bcdefg
Behrokh	S14	12.5b	25.7 ab	23.3 cdef
Behrokh	N14	15.4b	23.95 ab	15.1 ef
Reyhan	S7	12.25defg	18.95 bcdef	103.75 cdefg
Reyhan	N7	11.85defg	20.6 bcdef	124 bcdefg
Reyhan	S14	17.2b	29.5 ab	71.5ab
Reyhan	N14	13.6b	39.55a	16.4 ef
Mehr	S7	9.25efg	16.35cdef	95.2defg
Mehr	N7	16.65bcdef	17.05bcdef	134.8bcd
Mehr	S14	10.85b	32.43ab	10.35f
Mehr	N14	12.7b	17.6b	10.3f
Fajr	S7	7.75 fg	13.75ef	78.5efg
Fajr	N7	8.6efg	16.55cdef	152.4abcdef
Fajr	S14	11b	18.4b	65.1abc
Fajr	N14	19.16b	32.1ab	75.25a
Mahtab	S7	7.05g	12.45f	51g
Mahtab	N7	23abc	27.45abc	176abcd
Mahtab	S14	8.8b	29ab	10.85f
Mahtab	N14	14.55b	27.05ab	15.2ef

### Protein content

**Fig. 1:**
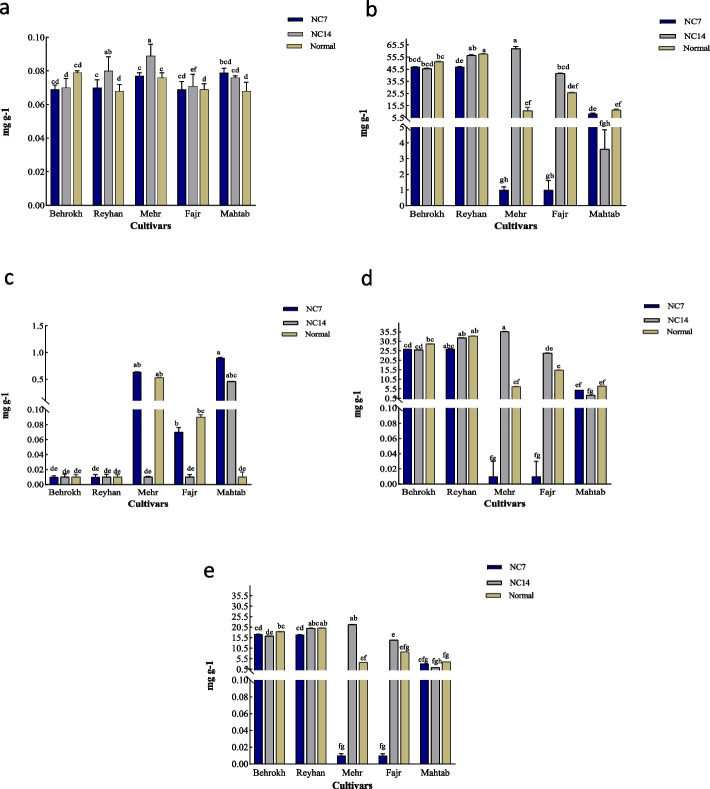
The effects of ND on five barley cultivars for protein content (**a**), total chlorophyll (**b**), carotenoid (**c**) chlorophyll b (**d**), and chlorophyll a (**e**) at 7 and 14 days after ND application. Values represent the means of three replications per treatment. Different letters demonstrate significant differences between treatments (*P* < 0.05, Duncan’s Multiple Range Test). NC7, 7 days after ND; NC14, 14 days after ND

Based on protein content measured at 7 and 14 days after ND application, the Behrokh and Mahtab cultivars did not show any significant differences from non-stressed condition. In the Reyhan cultivar, the protein content was significantly increased at 7 and 14 days after ND application as compared to non-stressed condition. However, the protein content was only significantly increased in the Mehr cultivar at 14 days after applying ND as compared to non-stressed condition. On the other hand, the protein content was significantly decreased in the Fajr cultivar at 14 days after applying ND as compared to non-stressed condition (Fig. 1a).

### Total chlorophyll content

Based on the total chlorophyll content, the Mahtab and Behrokh cultivars did not show any significant differences from non-stressed condition at 7 and 14 days after ND application. The Reyhan cultivar only showed a significant decrease in total chlorophyll content at 7 days after ND application. Further, as compared to non-stressed condition, the Mehr cultivar showed a significant decrease for total chlorophyll at 7 days and a significant increase at 14 days after ND application. The Fajr cultivar showed a significant increase in total chlorophyll content at 7 days, while it did not show any significant differences from non-stressed condition at 14 days after ND application (Fig. 1b).

### Carotenoids content

The Bahrokh and Reyhan cultivars showed no significant differences for carotenoid content at both time points of 7 and 14 days after ND application. The Fajr and Mehr cultivars showed no significant difference at 7 days after ND application; however, a significant decrease at 14 days after ND application was observed. The Mahtab revealed a significant increase both at 7 and 14 days after ND application (Fig. 1c).

### Chlorophyll b

Based on the content of chlorophyll b, the cultivars Bahrokh, Reyhan, and Mahtab did not show any significant differences as compared to non-stressed condition at 7 and 14 days after ND application. The Mehr cultivar showed no significant difference in chlorophyll b at 7 days after ND application but a significant increase at 14 days after ND application was observed. Compared to non-stressed condition, chlorophyll b was significantly reduced in the Fajr cultivar at 7 days after ND application; however, no significant difference from non-stressed condition was observed at 14 days after ND application (Fig. 1d).

### Chlorophyll a

Based on the content of chlorophyll a, the Mahtab and Fajr cultivars showed no significant differences as compared to non-stressed condition for both time points of 7 and 14 days after ND application. A significant decrease in chlorophyll a was observed at 7 days after ND application, while no significant difference from non-stressed condition was observed at 14 days after ND application in the Reyhan. The Mehr cultivar showed no significant difference at 7 days after ND application; however, a significant increase from non-stressed condition was observed at 14 days after ND application. The Behrokh revealed no significant difference at 7 days after ND application, but a significant decrease was observed at 14 days after ND application (Fig. 1e).

### Antioxidant enzymes’ content in leaves

#### APX enzymes in the leaves

**Fig. 2:**
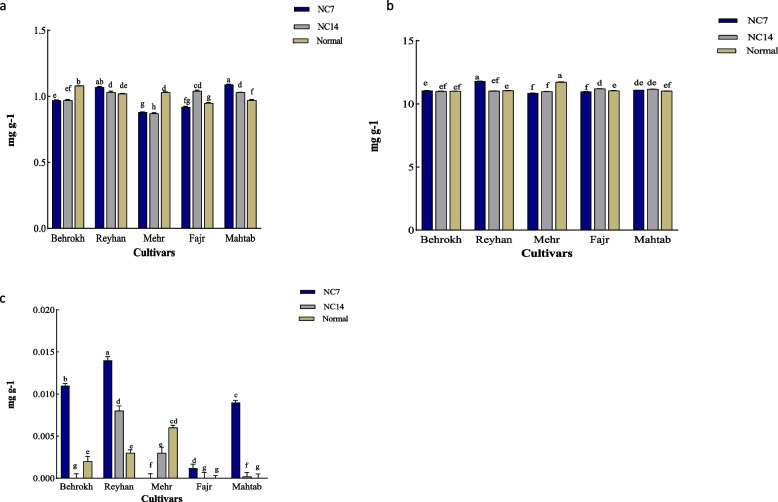
The effects of ND on APX (**a**), CAT (**b**), and POD (**c**) antioxidative enzyme activities in the leaves of barley cultivars under different concentrations. The error bars (mean ± SE) followed by various letters are statistically significant (*P* < 0.05, Duncan’s Multiple Range Test). Significant differences between the two concentrations are marked with different letters. NC7, 7 days after ND; NC14, 14 days after ND

APX enzymes in the leaves of the Mahtab cultivar were significantly increased as compared to non-stressed condition at both 7 and 14 days after ND application. A significant decrease was observed in the Behrokh and Mehr cultivars at 7 and 14 days after applying ND. At 7 days after applying ND, a significant increase in APX enzymes was observed in the Reyhan cultivar as compared to non-stressed condition. However, at 14 days after applying ND, no significant difference from non-stressed condition was observed. In the Fajr cultivar, a significant increase, as compared to non-stressed condition, was shown at 14 days after applying ND, but no significant difference was observed at 7 days after applying ND (Fig. 2a).

#### Catalase enzyme in leaves

In the Bahrokh and Mahtab cultivars, the amount of catalase enzyme did not show any significant differences at 7 and 14 days after applying ND when compared with non-stressed condition. A significant decrease from non-stressed condition was observed in the Mehr cultivar at two-time points of 7 and 14 days after applying ND. In the Fajr cultivar, catalase enzyme showed a significant decrease at 7 days after applying ND as compared to non-stressed condition, but at 14 days after applying ND, it showed a significant increase as compared to non-stressed condition. In the Reyahn cultivar, a significant increase as compared to non-stressed condition was observed at 7 days after ND application. But at 14 days after applying ND, no significant difference was observed in the content of catalase enzyme from non-stressed condition (Fig. 2b).

#### POD enzyme in leaves

In the Bahrokh cultivar, POD enzyme content was significantly increased as compared to non-stressed condition at 7 days after ND application. But at 14 days after ND was applied, a significant decrease in POD enzyme content was observed as compared to non-stressed condition. In the Reyhan and Mahtab cultivars, a significant increase in POD enzyme content compared to non-stressed condition was observed at 7 and 14 days after ND application. A significant decrease was observed in the Mehr at 7 and 14 days after ND application as compared to non-stressed condition. In the Fajr cultivar, a significant increase in POD content as compared to non-stressed condition was observed at 7 days after ND application. However, at 14 days after applying ND, no significant difference from non-stressed condition was observed (Fig. 2c).

### Antioxidant enzymes’ content in the root

#### APX enzymes in the root

**Fig. 3:**
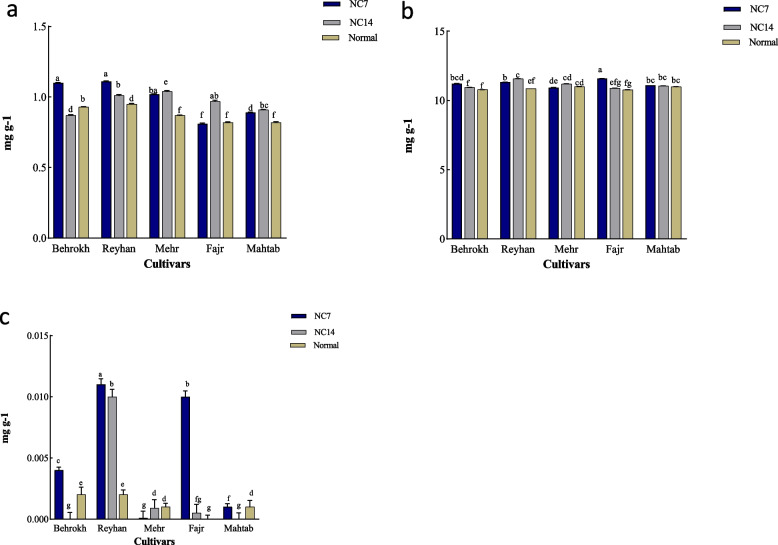
The effects of ND on APX (**a**), CAT (**b**), POD (**c**), and antioxidant enzyme activities in the root of barley cultivars under different concentrations. The error bars (mean ± SE) followed by various letters are statistically significant (*P* < 0.05, Duncan’s Multiple Range Test). Significant differences between the two concentrations are marked with different letters. NC7, 7 days after ND; NC14, 14 days after ND

In the Mahtab, Mehr, and Reyhan cultivars, a significant increase in the content of APX enzyme in the roots, as compared to non-stressed condition, was observed at 7 and 14 days after the application of ND. In the Behrokh cultivar, a significant increase as compared to non-stressed condition was observed at 7 days after ND application. However, at 14 days after applying ND, a significant decrease in the APX enzyme was observed as compared to non-stressed condition. In the Fajr cultivar, no significant difference from non-stressed condition was observed at 7 days after applying ND, but at 14 days after ND application, a significant increase was observed (Fig. 3a).

#### CAT enzymes in the root

The catalase content in the roots of Mehr and Mahtab cultivars was not significantly different from non-stressed condition at 7 and 14 days after ND application. In the Fajr and Bahrokh cultivars, a significant increase as compared to non-stressed condition was observed at 7 days after applying ND, but no significant difference was observed at 14 days after ND application. A significant increase as compared to non-stressed condition was observed in the Reyhan cultivar at 7 and 14 days after ND application (Fig. 3b).

#### POD enzymes in the root

The POD content in the roots of the Mahtab cultivar was observed to be significantly reduced from non-stressed condition at 7 and 14 days after ND application. In the Fajr cultivar, there was a significant increase in POD enzymes as compared to non-stressed condition at 7 days after applying ND. However, at 14 days after ND application, no significant difference from non-stressed condition was observed. A significant increase as compared to non-stressed condition was observed in the Reyhan cultivar at 7 and 14 days after ND application. In the Bahrokh cultivar, a significant increase in the content of POD enzyme in the roots as compared to non-stressed condition was observed at 7 days after ND application. But at 14 days after ND application, no significant difference from non-stressed condition was observed. In the Mehr cultivar, there was a significant decrease as compared to non-stressed condition at 7 days after applying ND. However, at 14 days after ND application, no significant difference in POD enzymes in the root, as compared to non-stressed condition, was observed (Fig. 3b).

### Gene expression in leaf

**Fig. 4:**
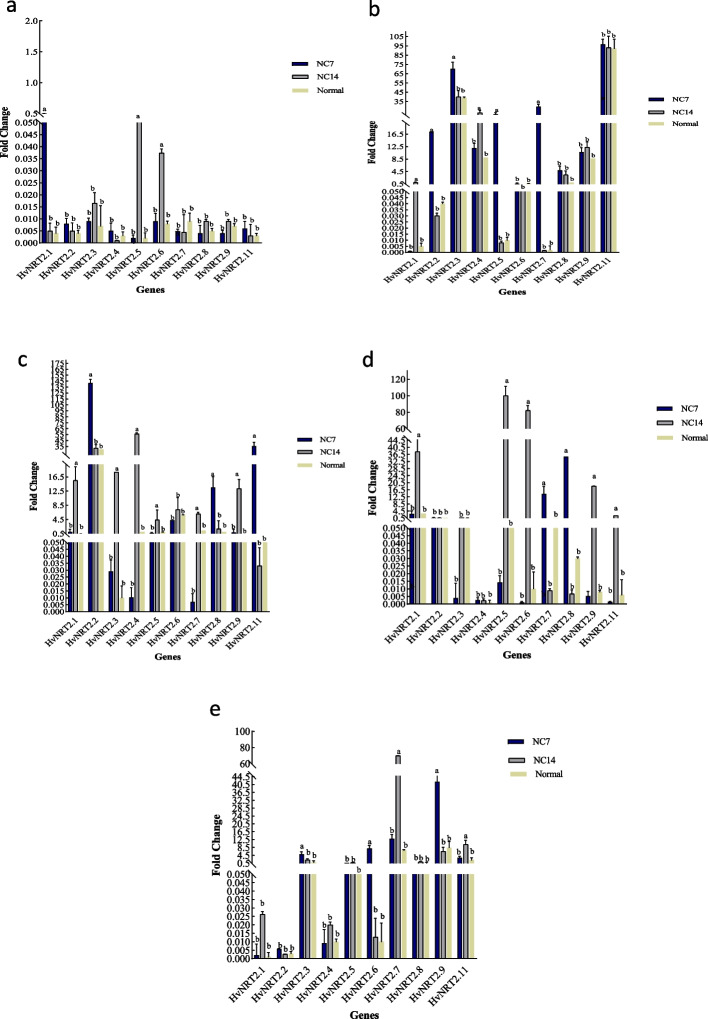
The qRT-PCR expressions of *HvNRT2.1*, *HvNRT2.2*, *HvNRT2.3*, *HvNRT2.4*, *HvNRT2.5*, *HvNRT2.6*, *HvNRT2.7*, *HvNRT2.8*, *HvNRT2.9*, and *HvNRT2.11* genes in leaf in five barley cultivars (Reyhan, Mehr, Mahtab, Fajr, and Behrokh) under ND conditions. Error bars represent the means ± SD taken from three independent biological replicates. NC7; 7 days after ND and NC14; 14 days after ND. Different lower case letters above columns indicate significant differences at the *P* < 0.05 between the non-stressed and ND conditions in the leaf. Duncan’s test was used to compare the treatment means at *P* < 0.05

The Reyhan cultivar did not show any significant differences at both two-time points of 7 and 14 days after ND application, except *HvNRT2.1*, *HvNRT2.5*, and *HvNRT2.6* genes (Fig. 4a). In the Mehr cultivar, the *HvNRT2.2*, *HvNRT2.3*, *HvNRT2.5*, and *HvNRT2.7* genes were more significantly expressed at 7 days after ND application as compared to non-stressed condition. The *HvNRT2.1* and *HvNRT2.4* genes showed higher significant expressions at 14 days after applying ND as compared to non-stressed condition (Fig. 4b). In the Mahtab cultivar, the *HvNRT2.2*, *HvNRT2.8*, and *HvNRT2.11* genes showed higher significant expressions at 7 days as compared to non-stressed condition. On the other hand, the *HvNRT2.1*, *HvNRT2.3*, *HvNRT2.4*, *HvNRT2.5*, *HvNRT2.7*, and *HvNRT2.9* genes were more significantly expressed as compared to non-stressed condition at 14 days after ND application (Fig. 4c). In the Fajr cultivar, the expression of *HvNRT2.7* and *HvNRT2.8* genes were significantly increased at 7 days after applying ND as compared to non-stressed condition. However, the *HvNRT2.1*, *HvNRT2.5*, *HvNRT2.6*, *HvNRT2.9*, and *HvNRT2.11* genes showed higher significant expressions at 14 days after applying ND as compared to non-stressed condition (Fig. 4d). In the Behrokh cultivar, the *HvNRT2.3*, *HvNRT2.6*, and *HvNRT2.9* genes showed higher significant expressions at 7 days after ND application as compared to non-stressed condition. Whereas, *HvNRT2.7* and *HvNRT2.11* exhibited higher significant expressions at 14 days after applying ND as compared to non-stressed condition (Fig. 4e).

### Gene expression in root

**Fig. 5:**
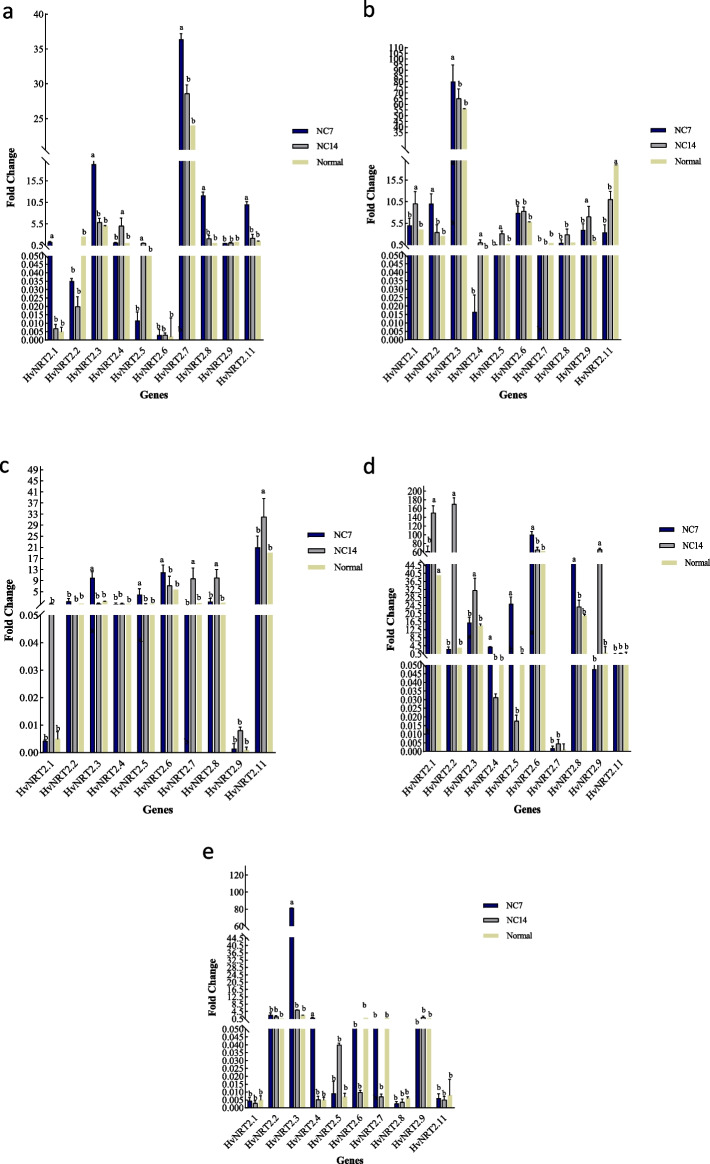
The qRT-PCR expressions of *HvNRT2.1*, *HvNRT2.2*, *HvNRT2.3*, *HvNRT2.4*, *HvNRT2.5*, *HvNRT2.6*, *HvNRT2.7*, *HvNRT2.8*, *HvNRT2.9*, and *HvNRT2.11* genes in leaves of five barley cultivars (Reyhan, Mehr, Mahtab, Fajr, and Behrokh) under ND condition. Error bars represent the means ± SD taken from three independent biological replicates. NC7; 7 days after ND and NC14; 14 days after ND. Different lower case letters above columns indicate significant differences at the *P* < 0.05 between the non-stressed and ND condition in root. Duncan’s test was used to compare the treatment means at *P* < 0.05

In the Reyhan cultivar, the *HvNRT2.1*, *HvNRT2.3*, *HvNRT2.7*, *HvNRT2.8*, and *HvNRT2.11* genes showed a significant increase in expressions at 7 days after applying ND as compared to non-stressed condition. The *HvNRT*2.4 and *HvNRT2.5* gene showed a significant increase at 14 days after applying ND as compared to non-stressed condition (Fig. 5a). In the Mehr cultivar, the expression of *HvNRT*2.2 and *HvNRT*2.3 were significantly increased at 7 days after ND application as compared to non-stressed condition, whereas, a significant increase in expressions of *HvNRT*2.1, *HvNRT*2.4, *HvNRT*2.5, and *HvNRT*2.9 were observed at 14 days after applying ND as compared to non-stressed condition (Fig. 5b). In the Mahtab cultivar, the *HvNRT* 2.3, *HvNRT*2.5, and *HvNRT*2.6 genes were significantly increased in expressions at 7 days after applying ND as compared to non-stressed condition. However, the expression of *HvNRT*2.7, *HvNRT*2.8, and *HvNRT*2.11 genes were significantly increased as compared to non-stressed condition (Fig. 5c). In the Fajr cultivar, the *HvNRT*2.4, *HvNRT*2.5, *HvNRT*2.6, and *HvNRT*2.8 were significantly increased at7 days after ND application, as compared to non-stressed condition. At 14 days after ND application, the *HvNRT*2.1, *HvNRT*2.2, *HvNRT*2.3, and *HvNRT*2.9 genes were significantly increased as compared to non-stressed condition (Fig. 5d). In the Behrokh cultivar, the *HvNRT2.3* and *HvNRT2.4* genes were significantly increased in expression at 7 days after ND application as compared to non-stressed condition (Fig. 5e).

### Principle component analysis

**Fig. 6:**
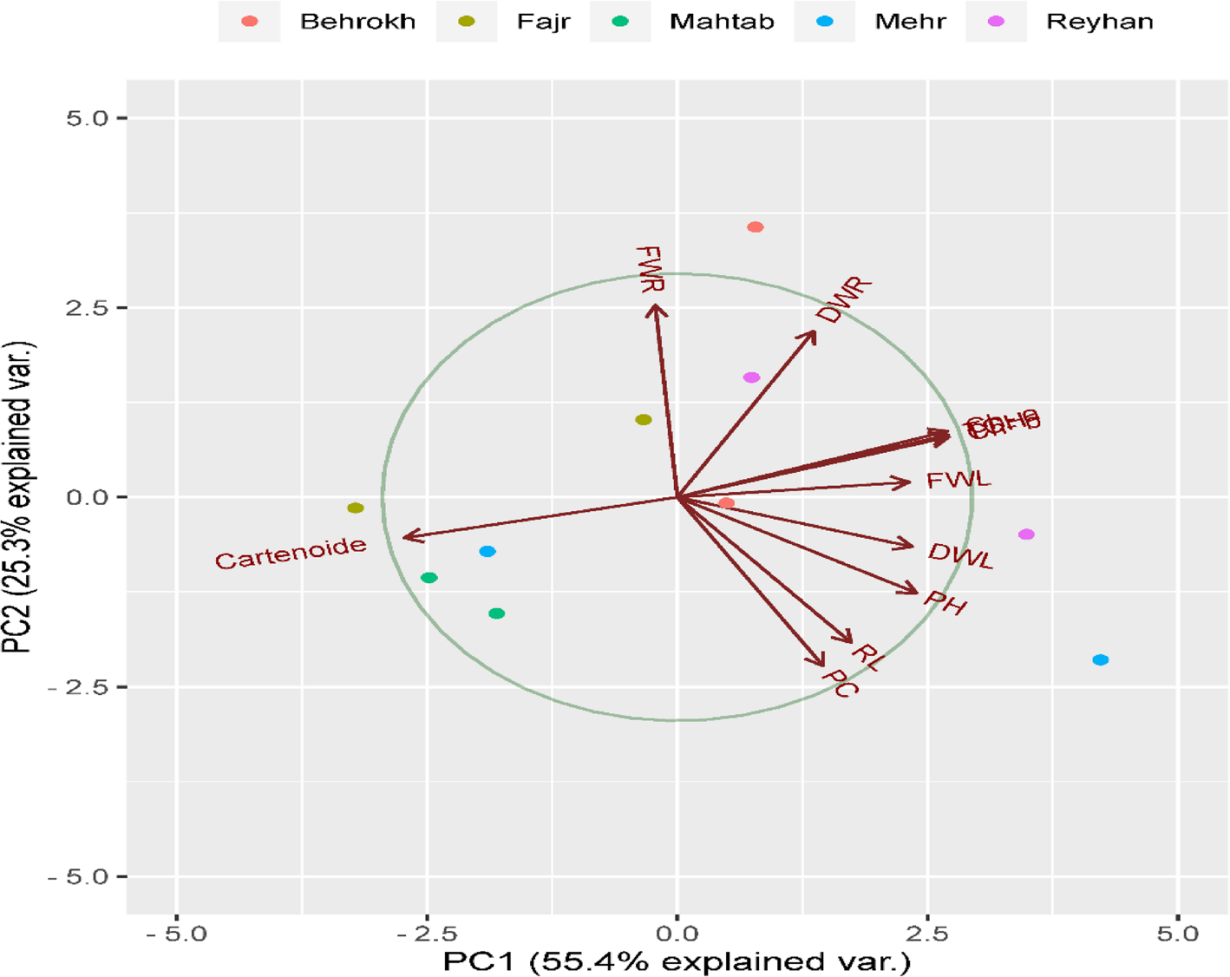
The principle component analysis of physiological and morphological traits among five barley cultivars under ND condition

The diversity of genotypes for morphological and physiological traits was evaluated using the bi-plot graphic display, and the cultivars were evaluated according to their principal component scores (PCA). The Reyhan, Fajr, and Behrokh cultivars are located on the upper right side of the graph and are considered as tolerant cultivars. The Mahtab and Mehr cultivars are located on the left bottom of the diagram and are considered as sensitive and semi-sensitive cultivars (Fig. 6).

### Correlation between measured traits and *HvNRT2* gene expression at 7 days after ND application

Investigating the correlation between traits and gene expression in roots and leaves at 7 days after applying ND showed that the HvNRT2.8r has a positive and significant correlation with carotenoid and HvNRT2.2r. Root dry weight had a positive and significant correlation with leaf dry weight. The HvNRT2.8r had a positive and significant correlation with HvNRT2.7l. There was a positive correlation between HvNRT2.7l and HvNRT2.7r. The HvNRT2.4r showed a positive and significant correlation with HvNRT2.5r, and HvNRT2.2r had a positive and significant correlation with HvNRT2.11r, HvNRT2.4l, HvNRT2.3r, and HvNRT2.3l. The HvNRT2.5l had a positive and significant correlation with HvNRT2.11l, HvNRT2.2l, HvNRT2.11r, HvNRT2.4l, HvNRT2.9r, HvNRT2.9l, HvNRT2.3r, and HvNRT2.3l.

A positive and significant correlation was observed between HvNRT2.11l and HvNRT2.2l, HvNRT2.11r, HvNRT2.4l, HvNRT2.9r, HvNRT2.9l, HvNRT2.3r, and HvNRT2.3l. The HvNRT2.2l had a positive and significant correlation with HvNRT2.11r, HvNRT2.4l, and HvNRT2.3r. A positively significant correlation between HvNRT2.11r and HvNRT2.4l, HvNRT2.3r, and HvNRT2.3l were observed. The HvNRT2.4l had a positive and significant correlation with HvNRT2.3r and HvNRT2.3l. A positive and significant correlation between HvNRT2.9r and HvNRT2.3l and between HvNRT2.9l and HvNRT2.3r and HvNRT2.3l were observed. The HvNRT2.3r had a positive and significant correlation with HvNRT2.3l.

**Fig. 7:**
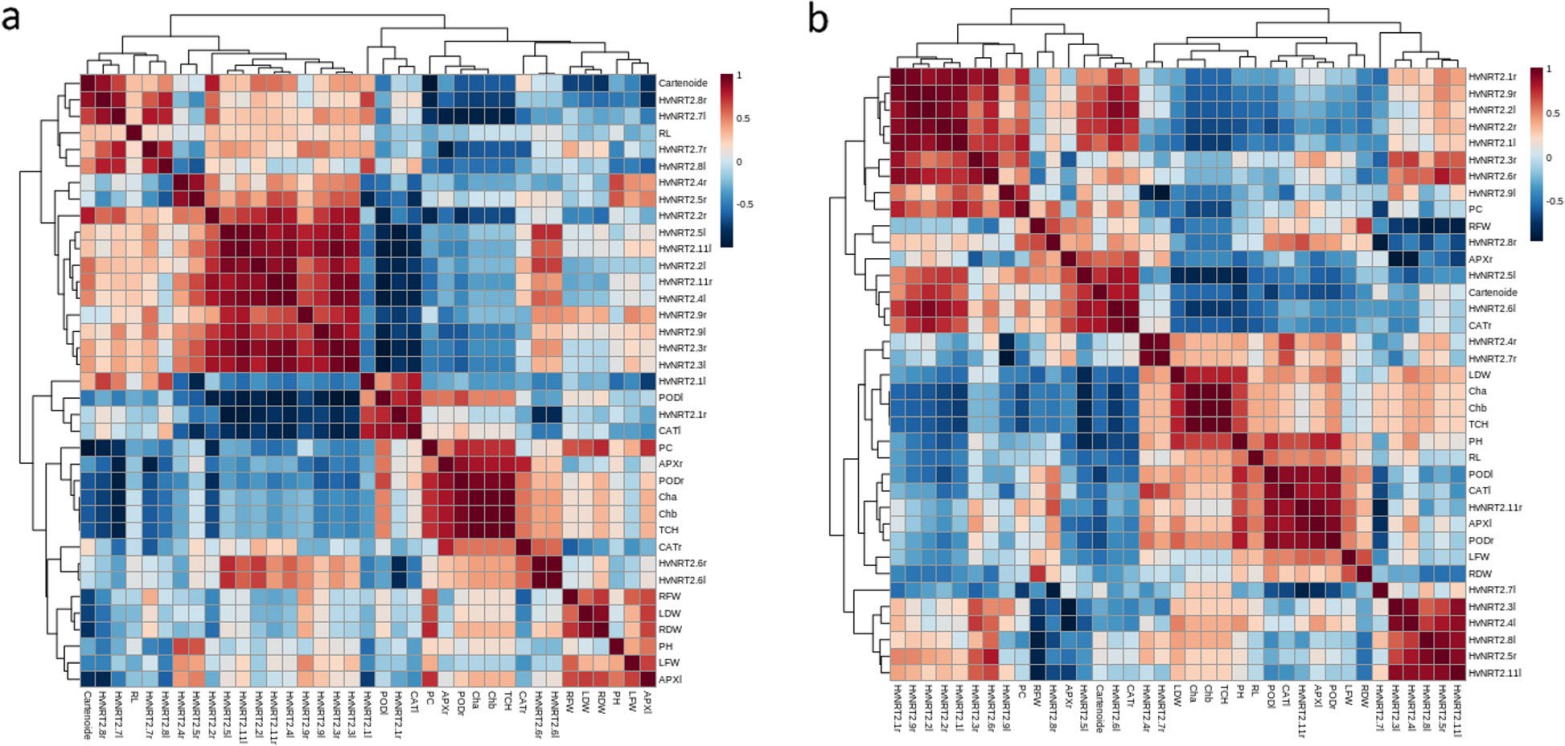
The correlation coefficients of physiological and morphological traits and gene expression at 7 (**a**) and 14 (**b**) days after ND application. Protein content (PC); plant height (PH); leaf dry weight (LDW); leaf fresh weight (LFW); root fresh weight (RFW); root dry weight (RDW); HvNRT2r (root HvNRT2); and HvNRT2l (leaf HvNRT2)

The HvNRT2.1l had a positive and significant correlation with CATl, whereas PODl and HvNRT2.1r had a positive and significant correlation with CATl. The APXr had a positive and significant correlation with PODr, Cha, Chb, and TCH. The PODr had a positive and significant correlation with Cha, Chb, and TCH. A positive and significant correlation between Cha and Chb and TCH was observed. The correlation between Chb and TCH was significantly positive. A positive and significant correlation between HvNRT2.6r and HvNRT2.6l was seen (Fig. 7a).

### Correlation between measured traits and gene expression at 14 days after ND

Investigating the correlation between traits and gene expression in roots and leaves at 14 days after applying ND showed that HvNRT2.1r has a positive and significant correlation with HvNRT2.9r, HvNRT2.2l, HvNRT2.2r, HvNRT2.1l, HvNRT2.3r, and HvNRT2.6r. The HvNRT2.9r had a positive and significant correlation with HvNRT2.6l, HvNRT2.2l, HvNRT2.2r, HvNRT2.1l, HvNRT2.6r, and HvNRT2.6l. A positive and significant correlation between HvNRT2.2l and HvNRT2.2r, HvNRT2.1l, HvNRT2.6r, HvNRT2.6l, and CATr were seen. The HvNRT2.2r showed a positive and significant correlation with HvNRT2.1l and HvNRT2.6l. A positive and significant correlation was seen between HvNRT2.1l and PC. The correlation between HvNRT2.3r and HvNRT2.6r was positively significant.

The HvNRT2.6r showed a positive and significant correlation with HvNRT2.5r. A positively significant correlation between HvNRT2.5l and HvNRT2.6l and CATr was observed. On the other hand, a negative and significant correlation between the HvNRT2.5l and Cha, Chb, TCH, and PH were observed. The carotenoid showed a positive and significant correlation with HvNRT2.6l and CATr. The correlation between HvNRT2.6l and CATr was positive, whereas the HvNRT2.4r revealed a positive and significant correlation with HvNRT2.7r. The Cha showed a positive and significant correlation with Chb and TCH. The Chb showed a significantly positive correlation with TCH. A positive correlation was observed between PH with PODr and between PODl with CATl, HvNRT2.11r, APXl, and PODr. Also, a positive correlation was observed between CATl and HvNRT2.11r, APXl, and PODr revealed. A positive correlation between HvNRT2.11r and APXl and PODr was observed. The APXl and PODr positively correlated. A positive correlation between HvNRT2.3l with HvNRT2.4l and HvNRT2.11l and between HvNRT2.4l and HvNRT2.5r and HvNRT2.11l were observed. The HvNRT2.8l showed a positive correlation with HvNRT2.5r and HvNRT2.11l and HvNRT2.5r revealed a positive correlation with HvNRT2.11l. A negative correlation was revealed between HvNRT2.11r with HvNRT2.7l. A positive correlation between CATr and HvNRT2.2l, HvNRT2.5l, carotenoid, and HvNRT2.6l were observed (Fig. 7b).

## Discussion

Nitrate is the main form of nitrogen utilized by most plants, acting both as an important nutrient and as a signal molecule involved in the metabolism, growth, development, and adaptation of plants to various stresses. In the present study, among the five cultivars (Reyhan, Behrokh, Fajr, Mehr, Mahtab), the Reyahn revealed higher values for root fresh weight (RFW), protein content, and antioxidant enzyme activity in root at two-time points of 7 and 14 days after ND as compared to non-stressed condition. Based on our results, the protein content only was significantly increased in the Reyhan cultivar at 7 and 14 days after applying ND, indicating that Reyhan had a high nitrogen use efficiency (NUE). Thus, the Reyhan cultivar can enhance protein content under ND and maintain nitrogen as a source for growth, biomass production, and seed yield. Based on the analysis of the expression of nitrate transporter genes among barley cultivars at 7 and 14 days after applying ND, the expression of HvNRT2.1 and *HvNRT2.4* genes were also significantly increased in the Reyhan. Under nitrogen-limited conditions, the NRT2 family plays a key role in acquiring NO^−^_3_ from the soil. The NRT2 include CmNRT2.1 in chrysanthemum [33], TaNRT2.1 in wheat [34, 35], and OsNRT2.1, OsNRT2.2, and their partner protein OsNAR2.1 in rice which play key roles in root NO^−^_3_ influx as main factors of HATS [24-1]. The *HvNRT2.1* and *HvNRT2.4* were strongly induced by NO^−^_3_ peaking at 7 and 14 days. Previous studies have shown that NRT2.1 under ND treatment can be a useful indicator to evaluate plant tolerance or sensitivity to ND [15]. Expression analysis of several NRT2 genes in response to nitrate indicates that ZmNRT2.1 and ZmNRT2.2 are the main genes controlling high-affinity nitrate uptake in maize [36]. At ND, the *HvNRT2.1*, *HvNRT2.2*, and *HvNRT2.4* genes had key roles in NO^−^_3_uptake and shoot and root growth. These findings have helped to detect candidate genes in response to ND. The contribution of *HvNRT2.1* and *HvNRT2.4* genes were required for optimal adaptation to N limitation in plants.

Breeding programs provide a possibility to improve the crop in predicted climatic conditions (for example, abiotic stress). In addition to morphological traits, other traits related to morphological such as physiological and biochemical traits play important roles in the selection of elite parents. Principal component analysis is a useful tool for screening tolerant genotypes [37]. Morphological traits can be used to identify superior parents to be utilized for germplasm introduction and classification [38, 39]. The results of principal components analysis and cluster analysis showed that the tolerant and sensitive cultivars were divided into separate groups with a similar pattern under ND. Thus, cultivars such as Reyhan and Fajr are considered as tolerant cultivars in response to nitrate stresses.

Chlorophyll degradation is closely related to reactive oxygen species (ROS). In the sensitive cultivar, Mahtab, chlorophyll content was decreased under ND conditions as compared to non-stressed. On the other hand, in the tolerant Reyhan cultivar, the chlorophyll content was higher under ND stress as compared to non-stress condition. Previous study has shown significant decreases in chlorophyll content in both sensitive and tolerant cultivars at 20d of low nitrogen [40]. Chlorophyll content decreased significantly under ND in creeping bentgrass [41]. Based on physiological traits, an increase in enzyme activity (CAT and APX activity) was observed, which contributes to the increase in AOX activity, especially in plants subjected to ND condition [42]. Plants can develop effective defense mechanisms against oxidative damage by inducing antioxidant enzymes [43, 44]. The CAT and POD are important antioxidant enzymes, playing important roles in minimizing the adverse effects of ROS.

Our results showed that under ND condition, the CAT, APX, and POD were increased in root and then in leaf, suggesting that enzyme activities are more active in the roots as compared in the leaves. According to Liao et al. [45], most enzyme antioxidants are more active in roots than in leaves [45]. Protein content is one of the main regulators of the osmotic potential of the cell. The presence of high protein content helps to maintain the osmotic potential of plant cells and their resistance against damage caused by stress [45, 46]. Our findings showed that RFW, protein content, and antioxidant enzymes were increased under ND condition, indicating the appropriate response of Reyhan to this stress. Previous studies have shown that appropriate N fertilization could increase antioxidant enzymes and physiological traits, improving plant tolerance in response to ND. Therefore, this promoted the accumulation of plant dry matter [47, 48].

Based on the data from five barely cultivars and correlation analysis, we found that the modifications of LDW had close relationships with the RDW and RFW, demonstrating that the root and shoot development could also be considered as useful indicators for the evaluation of plant ND response. In our study, Reyhan possessed higher RFW than other cultivars. Reyhan revealed a significant increase in antioxidant enzymes and protein content in leaves. In our finding, correlation analysis revealed that antioxidant enzymes and morphological and physiological traits at jointing were positively correlated under ND. It provides evidence for increasing tolerance by improving morphological and physiological stages.

Increasing the activity of antioxidant enzymes such as CAT and POD during stress is a defense mechanism in plants against free radicals, where the production of hydrogen peroxide is hindered and, as a result, prevents the degradation of plant proteins [49, 50]. Pervious study has shown that ND decreases CAT and POD activity in rice [51]. Some studies have shown that antioxidant enzyme was increased in response to ND in wheat (*Triticum aestivum*) leaves and (*Coffea arabica*) leaves under ND [52, 53]. Our findings revealed that the APX, POD, and CAT in the Rehyan’s root cultivar were increased significantly at 7 and 14 days after ND application. The Rehyan had a higher capacity to decompose H_2_O_2_ under ND condition. The Rehyan cultivar had a higher ability to remove H_2_O_2_ under a longer period of ND condition. Overall, our findings revealed that the tolerant genotype has a greater capacity than the sensitive genotype to remove H_2_O_2_ in shoots and roots under ND conditions. It has been reported that CAT activities were significantly increased in wheat leaves under low nitrogen [52]. As a result, ND led to oxidative damage in both cultivars, which was confirmed by increased activity of antioxidant enzymes and protein content. However, accessions suffered greater increases in leaf APX, root APX, root CAT, and root POD. The results enriched our understanding of antioxidant enzyme activity under ND condition, which will contribute to future research on revealing the molecular mechanisms associated with plant growth and survival under ND.

## Conclusion

In this study, five cultivars were screened in response to 7 and 14 days of ND application using morpho-physiological traits and gene expression of nitrate transporter genes. In conclusion, under ND, the *HvNRT2.1*, *HvNRT2.2*, and *HvNRT2.4* genes were induced in the leaf and root. Based on physiological traits, antioxidant enzymes and protein content were increased in response to ND in the Reyhan cultivar. Based on correlation between traits, there was a positive correlation between antioxidant enzyme in root and leaf HvNRT2.2, leaf HvNRT2.5, leaf HvNRT2.6, and carotenoid, indicating that the root had an important role under ND condition. The results of the principal components analysis showed that the tolerant and sensitive cultivars were placed in a group with almost similar patterns under ND condition. Further, cultivars such as Reyhan are considered as ND tolerant. Based on this, at 7 and 14 days after ND application, an increase in enzyme activity (POD, CAT, and APX) were observed, playing a significant role in antioxidant activity, especially in plants under ND condition. Therefore, the physiological and molecular mechanisms of *HvNRT2.4*, *HvNRT2.1*, and *HvNRT2.2* genes involved in NO^−^_3_ absorption and distribution, as well as adaptation to stress environments, such as ND, are worth further research. This study provides a basis for improving nitrogen application in different ND-tolerant cultivars and improving ND-tolerant cultivars in Iran.

## Data Availability

Not applicable

## Abbreviations

CAT: Catalase
POD: Peroxidase
APX: Ascorbate peroxidase
AOX: Antioxidant defense systems
ROS: Reactive oxygen species
